# LysM protein BdLM1 of *Botryosphaeria dothidea* plays an important role in full virulence and inhibits plant immunity by binding chitin and protecting hyphae from hydrolysis

**DOI:** 10.3389/fpls.2023.1320980

**Published:** 2024-01-08

**Authors:** He Zhang, Sheng-hui Wen, Pei-hang Li, Liu-yi Lu, Xu Yang, Chuan-jie Zhang, Li-yun Guo, Dongli Wang, Xiao-qiong Zhu

**Affiliations:** ^1^ Department of Plant Pathology and MARA Key Laboratory of Pest Monitoring and Green Management, College of Plant Protection, China Agricultural University, Beijing, China; ^2^ Sanya Institute of China Agricultural University, Sanya, China

**Keywords:** apple ring rot, agrobacterium-mediated infiltration, pathogen-associated molecular pattern (PAMP), lysin motif (LysM) effectors, pathogenicity

## Abstract

*Botryosphaeria dothidea* infects hundreds of woody plants and causes a severe economic loss to apple production. In this study, we characterized BdLM1, a protein from *B*. *dothidea* that contains one LysM domain. *BdLM1* expression was dramatically induced at 6 h post-inoculation in wounded apple fruit, strongly increased at 7 d post-inoculation (dpi), and peaked at 20 dpi in intact shoots. The knockout mutants of *BdLM1* had significantly reduced virulence on intact apple shoots (20%), wounded apple shoots (40%), and wounded apple fruit (40%). BdLM1 suppressed programmed cell death caused by the mouse protein BAX through *Agrobacterium*-mediated transient expression in *Nicotiana benthamiana*, reduced H_2_O_2_ accumulation and callose deposition, downregulated resistance gene expression, and promoted *Phytophthora nicotianae* infection in *N*. *benthamiana*. Moreover, BdLM1 inhibited the active oxygen burst induced by chitin and flg22, bound chitin, and protected fungal hyphae against degradation by hydrolytic enzymes. Taken together, our results indicate that BdLM1 is an essential LysM effector required for the full virulence of *B. dothidea* and that it inhibits plant immunity. Moreover, BdLM1 could inhibit chitin-triggered plant immunity through a dual role, i.e., binding chitin and protecting fungal hyphae against chitinase hydrolysis.

## 1 Introduction

*Botryosphaeria dothidea* is a fungal pathogen that infects hundreds of woody plants (Xiao et al., 2013; Marsberg et al., 2017). Apple ring rot caused by *B. dothidea*, also called white rot, is one of the most important diseases in apple production and has seriously affected the development of the apple industry in China (Guo et al., 2009). This pathogen commonly causes fruit rot, warts, rough skin, and cankers on apple stems (Li et al., 2009; Tang et al., 2012). With the release and availability of genome data (Liu et al., 2016; Marsberg et al., 2017; Wang et al., 2018; Hu et al., 2019; Liang et al., 2021; Rao et al., 2021; Yu et al., 2021) and recently improved gene disruption methods (Dong and Guo, 2020), research on gene function in *B. dothidea* is accelerating (Dong et al., 2021; Zhang et al., 2021).

During interactions of plants and pathogens, plants have evolved two layers of immune systems. The first layer involves cell-surface-localized pattern recognition receptors (PRRs), which recognize conserved pathogen-associated molecular patterns (PAMPs) to activate pattern-triggered immunity (PTI) (Jones and Dangl, 2006). This layer of the immune system is associated with a broad range of immune responses, including the generation of reactive oxygen species (ROS), the secretion of chitinases, and the induction of defense genes (Miya et al., 2007; Shimizu et al., 2010; Bozsoki et al., 2017). In contrast, pathogens secrete effectors to overcome PTI for successful colonization in hosts by perturbing host defenses (Boller and He, 2009). Plants have evolved a surveillance system to recognize these effectors and activate effector-triggered immunity, including hypersensitive cell death and defense-gene activation (Jones and Dangl, 2006; Wang et al., 2022). Over the past several decades, many typical PAMPs, such as fungal cell wall chitin, bacterial flagellar peptide flg22, and many effectors, including LysM motif-containing proteins, RXLR, and CFEM, have been characterized (Thomma et al., 2011; Wang et al., 2022).

LysM effectors are secreted proteins that do not carry any annotated domains, other than a different number of LysM domains; These domains are carbohydrate binding modules that appear in many prokaryotic and eukaryotic (Garvey et al., 1986; de Jonge and Thomma, 2009; Kombrink et al., 2017). Ecp6 was the first LysM effector characterized to contribute to the virulence of the tomato leaf mold pathogen *Cladosporium fulvum* (de Jonge et al., 2010). Later, it was found that LysM effectors also contribute to the virulence of many fungal pathogens, including the wheat *Zymoseptoria tritici/Mycosphaerella graminicola* pathogen (Mg1LysM and Mg3LysM), the rice blast fungus *Magnaporthe oryzae* (Slp1), the Brassicaceae anthracnose fungus *Colletotrichum higginsianum* (ChELP1 and 2), the vascular wilt fungal pathogen *Verticillium dahliae*, and the fruit pathogen *Penicillium* (Marshall et al., 2011; Mentlak et al., 2012; Lee et al., 2014; Takahara et al., 2016; Kombrink et al., 2017; Levin et al., 2017). According to several reports, LysM effector proteins competed to bind fungal cell wall chitin to prevent the elicitation of chitin-triggered host immunity and/ or protected hyphae from degradation by plant chitinases (Rovenich et al., 2016; Kombrink et al., 2017). For example, ChELp1 and ChELp2 of *C. higginsianum* and Ecp6 of *C. fulvum* suppress chitin-triggered defense responses by sequestering chitin fragments (de Jonge et al., 2010; Sánchez-Vallet et al., 2013). Mg1LysM and Mg3LysM of *M. graminicola* protect fungal hyphae against plant chitinase (Marshall et al., 2011). Mg3LysM of *M. graminicola* and Vd2LysM of *V. dahliae* could suppress chitin-triggered defense responses and protect fungal hyphae against hydrolysis by plant chitinase (Marshall et al., 2011). With a similar function, LysM effectors have been identified in mycoparasitism in insects (Cen et al., 2017) and have also been found to contribute to circumventing plant defense responses to facilitate arbuscular mycorrhizal symbiosis (Zeng et al., 2020). Although LysM effectors in many fungi have been widely characterized, little is known about their roles in woody fungal pathogens, including *B. dothidea*.

Previously, we identified five candidate LysM effectors in *B. dothidea* (Zhang et al., 2021). Here, we analyzed these proteins in *B. dothidea* using bioinformatics tools and studied the function of BdLM1 in *B. dothidea*. We analyzed the expression of BdLM1 during the infection process using qRT-PCR, tested the ability of BdLM1 to suppress programmed cell death and promote pathogen infection in *Nicotiana benthamiana* by infiltration, and further investigated the role of BdLM1 in vegetative growth and pathogenicity through gene disruption. The results of this study illustrated that BdLM1 plays a dual role in the interaction between *B. dothidea* and plants.

## 2 Materials and methods

### 2.1 Sequence analysis of LysM effectors in *Botryosphaeria dothidea*

Our previous study showed that there are five putative LysM effectors in *B*. *dothidea* (Zhang et al., 2021). Here, the structural domains of five putative LysM effectors were further analyzed using the NCBI Conserved Domain Search Tool (https://www.ncbi.nlm.nih.gov/Structure/cdd/wrpsb.cgi) (Bethesda, MA, USA). The SP was predicted using the online signalP-5.0 tool (http://www.cbs.dtu.dk/services/SignalP/) (DTU, Copenhagen, Denmark). Some LysM effectors from other fungi in JGI (https://genome.jgi.doe.gov/portal/) and GenBank were compared, and the phylogenetic tree was generated with MEGA 7.0 (Sudhir Kumar, Arizona State University, Knicks, AZ, USA) using the neighbor-joining method. A Poisson model was used for substitution of amino acids and pairwise deletion was used for gaps or missing data treatment. The statistical strengths were assessed by bootstraps with 1000 replicates.

### 2.2 Functional verification of signal peptides

To confirm the secretion activity of BdLM1, a yeast secretion trap assay was used following the description by Zhang et al. (2021). Specifically, fusion of the predicted SP of BdLM1 to the N-terminal of the secretion-defective invertase gene (*suc2*) in the vector pSUC2, was transformed into yeast strain YTK12 using a T2001Frozen-EZ Yeast Transformation II Kit (Zymo Research, Irvine, CA, USA). YTK12 was cultured on yeast minimal tryptophan dropout medium (CMD-W medium, 0.67% yeast N base without amino acids, 0.075% tryptophan dropout supplement, 2% sucrose, 0.1% glucose, and 2% agar) and YPRAA medium (1% yeast extract, 2% peptone, 2% raffinose, and 2 µg of antimycin A per liter). The coding sequences of the SP of Avr1b and the first 25 amino acids of Mg87 were used as the positive and negative controls, respectively. The primers for vector construction were listed in **Table S1**.

### 2.3 *Agrobacterium tumefaciens*-mediated infiltration assay in *N. benthamiana*

To determine whether BdLM1 regulated the plant immune response, an *A*. *tumefaciens*-mediated infiltration assay in *N*. *benthamiana* was performed using a previously described method (Zhang et al., 2021). Both the ORF (without SP) sequences and the full coding gene of *BdLM1* were amplified from the cDNA of *B*. *dothidea* isolates ZY7 or HTLW03 and cloned into the plasmid pGR107 with a 3× flag-tag fused at the N-terminus using the ClonExpress II One-Step Cloning Kit (Vazyme, Nanjing, China), according to the manufacturer’s instructions. After verification using PCR with the pGR107-F/R primers (**Table S1**) and sequencing, the generated construct was then transformed into *A*. *tumefaciens* strain GV3101 by electroporation.

The assays of *A. tumefaciens*-mediated transient gene expression in *N. benthamiana* were performed using a previously described method (Zhang et al., 2021). Specifically, *A. tumefaciens* cells carrying BdLM1 were cultivated overnight in a Luria–Bertani medium containing 50 mg/mL kanamycin and rifampicin in a shaker at 28°C and 180 rpm. The *A*. *tumefaciens* cells were harvested, washed three times, and then resuspended in infiltration buffer to a final OD_600_ of 0.5. After being kept at room temperature for 3 h, the *A*. *tumefaciens* cells carrying BdLM1 were initially infiltrated via needleless syringes into the leaves of 4–6-week-old *N*. *benthamiana* plants. A total of 15 leaves from five tobacco plants, were used. Infiltrations of buffer and *A*. *tumefaciens* cells carrying pGR107-GFP were used as the negative controls. After 24 h of initial infiltration, the same infiltration site was challenged with *A*. *tumefaciens* cells carrying BAX. The entire assay was repeated at least once. Cell death symptoms on infiltrated leaves were observed and photographed 6 d after initial infiltration. Western blotting was performed as described by Zhang et al. (2021).

To determine the immune response of the plant, infection by *P*. *nicotianae* was further tested after BdLM1 infiltration in *N*. *benthamiana* using a previously described method (Wang et al., 2019; Yang et al., 2019). In brief, the ORF of BdLM without SP was amplified from the cDNA of *B*. *dothidea* isolate HTLW03, cloned into the plasmid pSuper with a GFP-tag or the plasmid pGR107-GFP, and transformed into *A*. *tumefaciens* strain GV3101 by electroporation. Totally, 12 N. *benthamiana* leaves were collected 36 h after agroinfiltration and kept on filter paper with sterile double-distilled H_2_O in Petri dishes, and the plates were kept in plastic boxes. The infiltrated region was inoculated with a *P. nicotianae* mycelial plug (0.5 mm in diameter). The lesion was photographed at 60 hpi and the area was measured. Total DNA was extracted from leaf disks (3 cm in diameter) at infection sites 60 hpi with *P*. *nicotianae*. The biomass of *P*. *nicotianae* in inoculated leaves was determined with quantitative PCR (qPCR) using the *N. benthamiana actin* gene and the *P*. *nicotianae* elongation factor (*EF1α*) gene as internal controls (**Table S1**). The H_2_O_2_ content in *N*. *benthamiana* leaves was tested at 12 hpi with *P*. *nicotianae* after being infiltrated with GFP or BdLM1, using a previously described method (Wang et al., 2019; Yang et al., 2019). In addition, callose deposition and the expression of the PR protein were assayed at 48 hpi, as previously described (Dong et al., 2021). *NbPR1* and *NbNPR1* were determined with qPCR using the elongation factor (*EF1α*) gene as an internal control (**Table S1**). The results of qPCR were analyzed using the 2^−ΔΔct^ method (Livak and Schmittgen, 2001). The experiment contained three replicates. The assay was repeated once.

### 2.4 Confocal microscopic analysis

To study the subcellular localization of BdLM1 in plants, BdLM1 was cloned and inserted into the pCAM35s-GFP plasmid at the *Xba* I and *Sal* I sites with the primers listed (**Table S1**), generating the fusion vector pCAM35s-GFP-BdLM1. The construct pCAM35s-GFP-BdLM1 and the empty vector pCAM35s-GFP were transformed into *Agrobacterium* strain GV3101 and then infiltrated into the leaf epidermis of *N*. *benthamiana*. At 60 hpi, *N*. *benthamiana* leaf pieces (0.2 × 0.2 cm in size) were mounted in water on glass slides for observation. The fluorescence was imaged using a Leica TCS SP8 confocal microscopy system (Leica, Wetzlar, Germany). GFP fluorescence was excited using 488- and 552-nm laser lines.

### 2.5 RNA extraction and qRT-PCR analysis

To detect the expression pattern of *BdLM1* in apple during infection by *B*. *dothidea*, the mycelium and fruit tissues (2 × 2 cm) were collected from 36 inoculation sites at 0, 6, 12, 24, 36, 48, and 72 hpi. Similarly, the mycelium and bark tissues (0.5 × 0.5 cm) from 18 inoculation sites were collected at 0, 1, 3, 7, 20, and 30 dpi. The total RNA of each sample was extracted using EASY spin plus plant RNA extraction kit (Aidlab Biotech. Beijing, China). The purity and concentration of RNA were checked using a Nanodrop 2000 Spectrophotometer (Thermo Fisher Scientific, Waltham, MA, USA). First-strand cDNA was synthesized using Reverse Transcriptase M-MLV (Takara, Dalian, China) following the manufacturer’s instructions. The *B*. *dothidea actin* gene was used as an internal control. PCR was performed in qPCR Tower 2.0 (Analytik, Jena, Germany) using TB Green Premix DimerEraser™ qPCR mix (Takara, Dalian, China), with primers listed in **Table S1**. Relative expression values were calculated using the 2^−ΔΔCt^ method (Livak and Schmittgen, 2001). Means from three replicates were used. The experiments were repeated once with a different set of biological samples.

### 2.6 Generation of gene deletion and complementary transformants

For gene deletion and complementation, polyethylene glycol (PEG)-mediated homologous recombination was performed as previously described by Dong and Guo (2020). Specifically, we constructed a gene homologous recombination (GHR) plasmid containing a hygromycin resistance gene (*hph*) with flanking sequences of *BdLM1*. The 5’ and 3’ flanking fragments of size 1000 bp were amplified from the genomic DNA of *B*. *dothidea* HTLW03. The two fragments were ligated to the 5’ and 3’ ends of the 1800 bp *hph* gene and introduced into the pMD19-T vector using the ClonExpress II One Step Cloning Kit (Vazyme, Nanjing, China). The recombinant plasmid was introduced into *B. dothidea* HTLW03 protoplasts using PEG. The generated gene deleted transformants were verified with PCR using the primer pairs listed in **Table S1** and a Southern blot. The complementary fragment of BdLM1, including approximately 1600 bp promoter, the ORF, and 500 bp terminator, was amplified from the genomic DNA of *B*. *dothidea* and inserted into the pMD19-T-NEO plasmid at the *Hin*d III site. The generated transformants were verified by phenotype characteristics.

### 2.7 Morphological characteristics and pathogenicity assay

Mycelial plugs (5 mm in diameter) of the WT strain HTLW03 and its transformants from the edge of a growing colony were transferred to new PDA plates. Three replicated plates per strain were used and incubated at 26°C in the dark for 48 h. Colony characteristics were examined, and the colony diameter was measured. Furthermore, melanin was observed after incubation for 5 and 10 d.

To induce conidia formation, the aerial mycelia of 3-d-old colonies on PDA medium were scraped off with a scalpel and incubated at 26°C under a near-UV light for 10 d. The mature pycnidia were collected in a 1.5 mL microcentrifuge tube with 0.5 mL of sterile ddH_2_O and crushed with a pestle. The concentration of the conidia suspension was measured with a hemocytometer. The length and width of 50 conidia per isolate were measured under a compound microscope (Olympus Model BX41TF). In addition, conidial germination was tested on water agar at 26°C in the dark for 4–5 h. The percentage of conidial germination was estimated by examining 100 conidia per replicate, with three replicates for each isolate. Each experiment was repeated once.

The pathogenicity of the WT and its transformants was tested on intact shoots, wounded shoots and fruit of apple (*Malus domestica* Borkh. ‘Fuji’) as previously described (Dong et al., 2021; Tang et al., 2012; Zhang et al., 2021). Symptoms on intact apple shoots were observed and the severity of the disease was recorded at 30 dpi as described by Dong et al. (2021). The length of the lesion on wounded shoots was measured at 5–7 dpi, and the diameters of the lesion on wounded apple fruit were measured at 2 dpi. Each experiment included three apple fruits or five shoots. The pathogenicity test was repeated once.

### 2.8 Heterologous protein production in *Escherichia coli*

Prokaryotic expression of BdLM1 was performed as described by Tian et al. (2021), with some modifications. Specifically, the opening reading frame of *BdLM1* amplified with primers listed in **Table S1**, was ligated into the pET-SUMO vector with a 6 × His-tag, and transformed into *E*. *coli* BL21 (DE3) pLysS (ZOMANBIO, Beijing, China). BdLM1 expression was induced with 0.5 mM isopropyl β-D-1-thiogalactopyranoside at 26°C for 20 h. After *E*. *coli* cells were harvested through centrifugation at 7500 rpm for 10 min, the precipitate was resuspended in 50 mL cell lysis buffer (50 mM Tris-HCl pH 8.0, 150 mM NaCl, 20 mM imidazole), incubated at 4°C for 15 min with stirring, and centrifuged at 12,000 rpm for 25 min. The resulting cleared supernatant was immediately placed on ice for further purification.

For the purification of BdLM1, His60 Ni Superflow resin (Clontech, Mountain View, CA, USA) was used. After being equilibrated with wash buffer (50 mM Tris-HCl pH 8.0, 150 mM NaCl, and 20 mM imidazole), the protein preparation was loaded onto the column. The target protein was eluted with elution buffer (50 mM Tris-HCl pH 8.0, 150 mM NaCl, and 500 mM imidazole), and the purity of the elution was tested on a sodium dodecyl sulfate polyacrylamide gel electrophoresis (SDS-PAGE) gel, followed by Coomassie brilliant blue staining. Furthermore, the protein of the elution was concentrated to the required concentration.

### 2.9 Chitin binding assay

The assay was performed as described by Tian et al. (2021), with some modifications. In brief, 500 µL of protein solution containing 30 μg/mL *E*. *coli*-produced BdLM1 protein was incubated with 5 mg chitin, chitosan, cellulose, or xylan (Yuanye, Shanghai, China) in a 100 rpm shaker at 4°C for 6 h. The samples were centrifuged at 13,000 g for 5 min. The supernatants were collected and concentrated to a volume of approximately 100 µL. The pellets were washed three times with incubation buffer, and then resuspended in 100 µL demineralized water. Then, 50 µL of the pellet solution or the supernatant were individually incubated with 50 µL of SDS-PAGE protein loading buffer (2×; 200 mM Tris-HCl, pH 6.5, 0.4 M dithiothreitol, 8% sodium dodecyl sulfate, 6 mM bromophenol blue, and 40% glycerol) at 95°C for 10 min. Samples were analyzed with a Western blot using anti-His antibodies. Photos were taken using Azure Biosystems (Azure, Dublin, CA, USA) in a custom setting.

### 2.10 Reactive oxygen species measurement

ROS production measurements were performed as described by Tian et al. (2021). For each treatment, four *N*. *benthamiana* leaf disks (Ø = 0.5 cm) from 2-week-old *N*. *benthamiana* plants, were placed into a 96-well microtiter plate, and rinsed with 200 µL fresh demineralized water for 24 h. Replaced water by 50 µL fresh demineralized water, the plate was incubated for 2 h at room temperature. Meanwhile, mixtures of (GlcNAc)6 (Sigma-Aldrich, St. Louis, MO, USA) and the BdLM1 protein were incubated for 2 h. Then (GlcNAc)6 was added to a final concentration of 40 µM in the absence or presence of 50 µM BdLM1 protein in a measuring solution containing 200 µM luminol (Biotopped, Beijing, China) and 20 µg/mL horseradish peroxidase (Biotopped, Beijing, China). Similarly, flg22 was added to a final concentration of 1 µM in the absence or presence of 50 µM BdLM1. Chemiluminescence were measured every 1 min over 40 min in a Tecan Infinite F200 Microplate Reader (Tecan, Männedorf, Switzerland).

### 2.11 Hyphal protection against chitinase hydrolysis

The assay was performed using a previously described method (Tian et al., 2021). Specifically, *Fusarium oxysporum* f. sp. *lycopersicum* conidia were harvested from a 4-d-old culture on CMC medium (15 g of Carboxymethyl cellulose, 1 g of NH_4_NO_3_, 1 g of KH_2_PO_4_, 0.5 g of MgSO_4_·7H_2_O, 1 g of Yeast Extract) filtrated with Miracloth (Merck, KgaA Darmstadt, Germany), and adjusted to a concentration of 10^6^ spores/mL with potato dextrose broth. Conidia suspensions in aliquots of 50 µL were incubated overnight at room temperature. BdLM1 protein was added to a final concentration of 20 µM. After 2 h of incubation, 2 µL of chitinase from *Streptomyces griseus* (Yuanye, Shanghai, China) was added to the appropriate wells. Sterile water was added as a control. Further incubated for 4 h, hyphal growth was inspected with an Olympus BX41 microscope.

### 2.12 Statistical analysis

Statistical analysis of the data in this study was performed using Microsoft Office and SPSS (IBM, Armonk, NY, USA). To determine whether the effects of treatment were statistically significant, an analysis of variance was first conducted. When treatment effects were significant, multiple mean comparisons were performed using Duncan’s test with a confidence level of 0.05.

## 3 Results

### 3.1 BdLM1 in *B. dothidea* is a typical LysM protein with secretion activity

**Figure 1:**
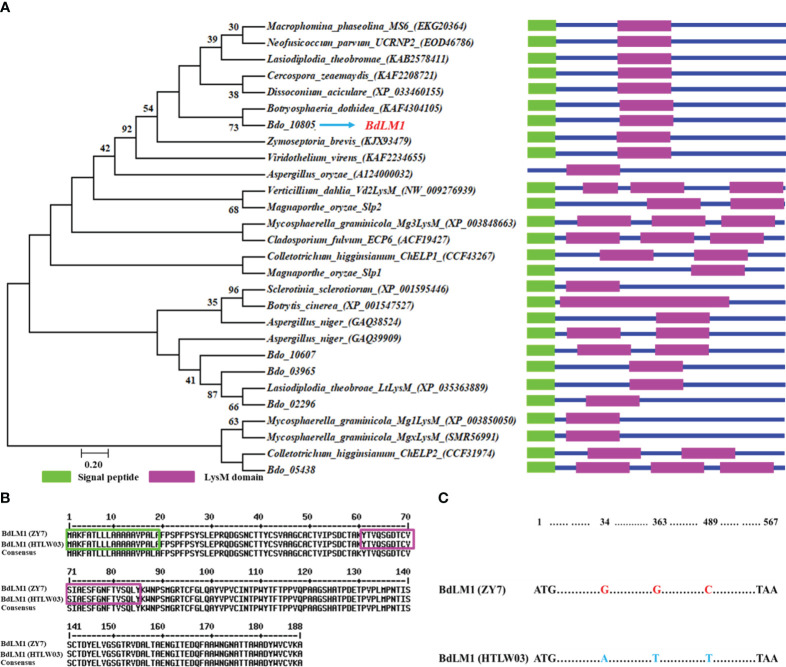
Bioinformatic analyses of the candidate effector containing the LysM domain and BdLM1 in *Botryosphaeria dothidea*. **(A)** Phylogenetic tree of LysM proteins. The amino acid sequences of LysM proteins of fungi were obtained from the NCBI database and used to generate the phylogenetic tree using MEGA 7 with the neighbor-joining method (1000 replicates). The LysM domain was predicted using CDD/SPARCLE (https://www.ncbi.nlm.nih.gov/Structure/cdd/wrpsb.cgi). **(B)** Amino acid sequence comparison of BdLM1 in HTLW03 and ZY7 strains using MultAlin (http://multalin.toulouse.inra.fr/multalin/multalin.html). Signal peptide prediction was performed with the SignalP 5.0 Server (http://www.cbs.dtu.dk/services/SignalP/). The green box indicates the putative BdLM1 signal peptide while the pink box indicates the putative LysM domain. **(C)** Nucleotide acid sequence comparison of *BdLM1* in the HTLW03 and ZY7 strains.

Our previous study showed that there were five proteins containing the LysM domain in *B*. *dothidea* (Zhang et al., 2021). Here, we first compared the LysM proteins from *B*. *dothidea* with those from other plant pathogenic fungi. Phylogenetic analysis showed that the five candidate LysM proteins from *B*. *dothidea* (Bdo_02296, Bdo_03965, Bdo_10607, Bdo_10805, and Bdo_05438) were divided into four groups (**Figure 1A**). Bdo_10805, which contained 189 amino acids and a LysM motif, was designated as BdLM1. It clustered into a group different from those well-known effectors including Ecp6, Slp1, ChELp1, and Vd2LysM (**Figure 1A**). BdLM1 had a signal peptide (SP) of 19 amino acids in the N-terminal, suggesting that it may be a secreted protein, and the amino acids from 61 to 95 constituted a typical LysM domain (**Figures 1A, B**). In addition, the amino acid sequences of BdLM1 from isolates ZY7 and HTLW03 were identical (**Figure 1B**), despite the difference of three nucleotide acids (**Figure 1C**).

**Figure 2:**
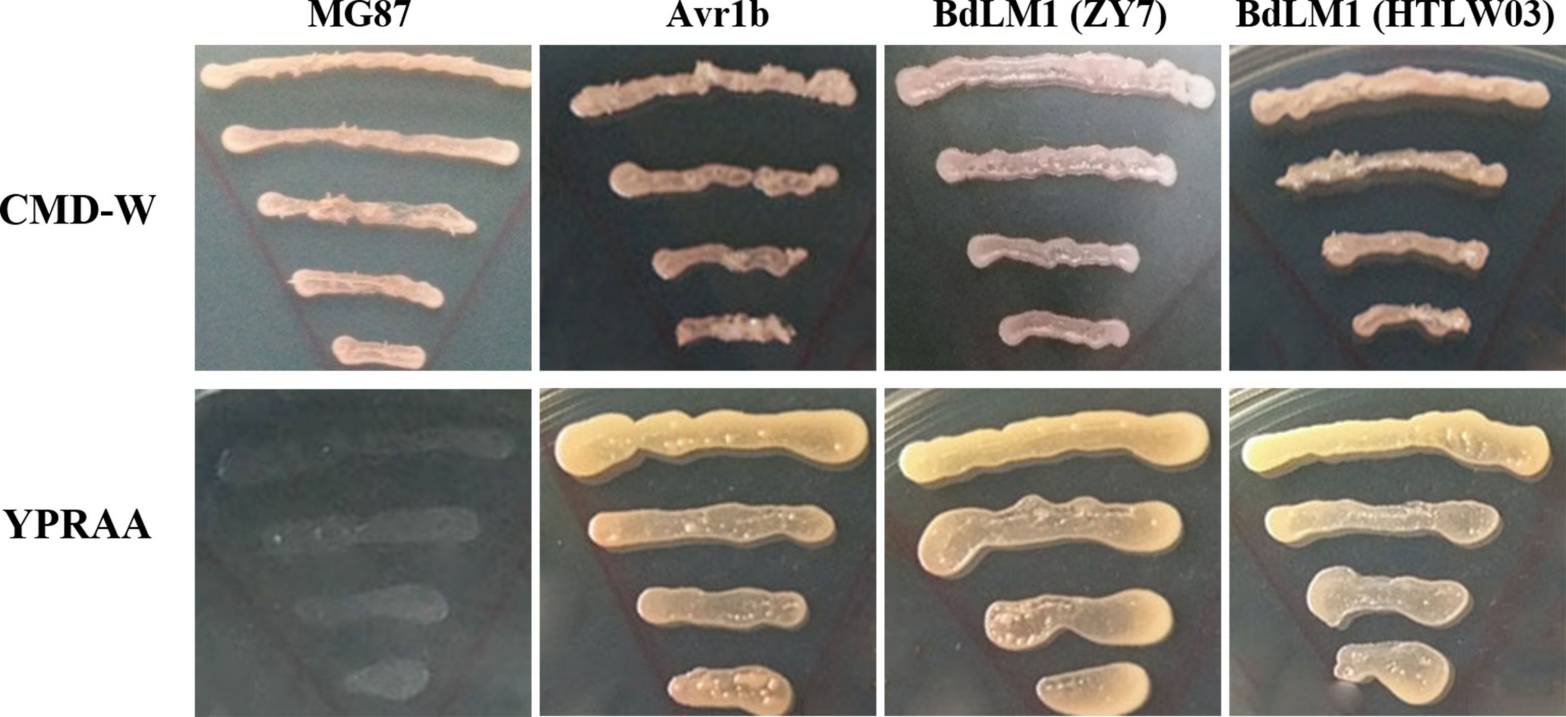
Yeast invertase secretion assay of the predicted signal peptide of BdLM1. The signal peptide sequences of PsAvr1b and MG87 were used as the positive and negative controls, respectively, to assay the predicted signal peptide of BdLM1. CMD-W (minus Trp) plates were used to select yeast strain YTK12 carrying the pSUC2 vector. YPRAA media were used to indicate invertase secretion.

As BdLM1 was predicted to have a SP, we tested its secretory activity using the yeast secretion trap assay described by Zhang et al. (2021). The SPs of BdLM1 from either HTLW03 or ZY7 could restore the growth of invertase-deficient yeast on YPRAA medium, similar to the positive control Avr1b (**Figure 2**). These results suggest that the SPs of BdLM1 can guide the secretion of the truncated invertase and that BdLM1 has secretory activity.

### 3.2 High BdLM1 expression during *B. dothidea* infection of apple

**Figure 3:**
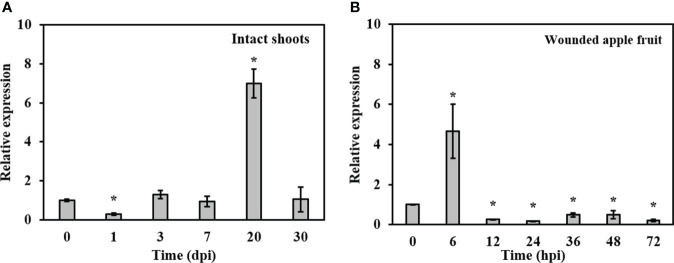
Relative expression levels of *BdLM1* in *Botryosphaeria dothidea* during the infection stages. **(A)** Expression in intact apple shoots. The shoot tissues inoculated with wild type HTLW03 were harvested at 0, 1, 3, 7, 20, and 30 d post-inoculation (dpi) for RNA extraction. **(B)** Expression in wounded apple fruit. The fruit tissues inoculated with wild-type HTLW03 were harvested at 0, 6, 12, 24, 36, 48, and 72 h post-inoculation (hpi) for RNA extractions. The relative transcript levels of *BdLM1* at different time points after inoculation were normalized by the actin gene and calibrated against that of mycelia. The relative transcript level of BdLM1 was calculated using the 2^-ΔΔCT^ method. The assays were performed with two independent biological repetitions and three replicates each. Error bars represent the standard error. Asterisks indicate statistical significance according to the Student’s t-test (*P < 0.05).

To examine the expression profile of *BdLM1* during the infection of *B*. *dothidea*, we extracted RNA from apple shoots or fruit at various times post-inoculation and quantified its expression using qRT-PCR. In intact shoots, *BdLM1* expression was low at 1 d post-inoculation (dpi), strongly increased at 7 dpi, peaked at 20 dpi, and decreased at 30 dpi (**Figure 3A**). In wounded apple fruit, *BdLM1* expression was highest at 6 h post-inoculation (hpi) and significantly decreased to low levels at 12, 24, and 72 hpi (**Figure 3B**). These results indicate that *BdLM1* plays a crucial role in the infection process of *B*. *dothidea*.

### 3.3 BdLM1 gene is important for the vegetative growth and virulence of *B. dothidea*

**Figure 4:**
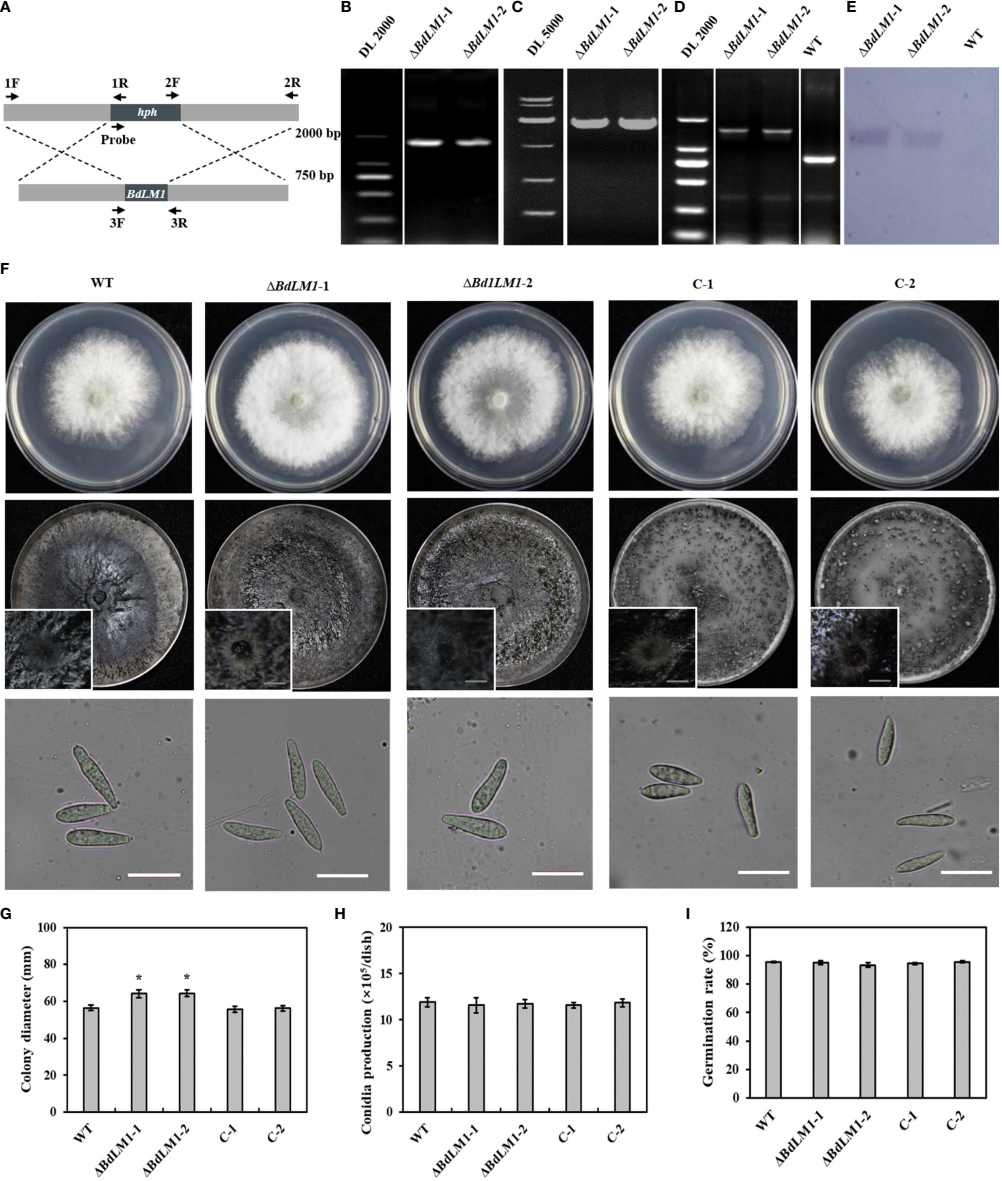
Homologous recombination strategy, verification, colony morphology, and conidial characteristics of *BdLM1* knockout transformants. **(A)** Homologous recombination strategy and location of primers used to verify *BdLM1* knockout transformants. **(B–E)** PCR and Southern blot analysis of *BdLM1* knockout transformants. **(B)** Approximately 1.6 kb PCR products upstream using primer F1/R1. **(C)** Approximately 1.9 kb PCR products downstream using primer pair F2/R2. **(D)** Using primer pair F3/R3, approximately 0.8 kb PCR product was amplified from wild type isolate HTLW03, and 1.3 kb PCR products were obtained from the knockout transformants. **(E)** Southern blot of the two knockout transformants and wild type isolate HTLW03. The 500 bp *hph* gene fragment was used as a probe in Southern blot analysis. **(F)** The wild type, *BdLM1* gene deletion mutants, and two complementary transformants were cultured on potato dextrose agar (PDA) at 25°C in the dark. Photographs were taken at 2 d post-culture, Bars = 10 μm. **(G–I)** Statistical results of colony diameters, conidia production, and germination rate, respectively. Data were the averages (and standard errors) of the values from two independent experiments. The asterisk indicates the significant difference according to the Student’s t-test (*P <0.05).

In order to determine the biological function of *BdLM1* in *B*. *dothidea*, we generated the *BdLM1* knockout transformants by homologous recombination as previously described by Dong and Guo (2020) (**Figure 4A**). *BdLM1* knockout transformants were identified using polymerase chain reaction (PCR). As expected, PCR products of approximately 1.6, 1.9, and 1.6 kb for upstream (with 1F and 1R), downstream (with 2F and 2R), and ORF fragments (with 3-F and 3-R), respectively, were amplified (**Figures 4B–D**). In Southern blotting using a hygromycin B phosphotransferase (*hph*) gene probe, the WT showed no hybridization signal, while the two knockout transformants showed a unique hybridization band (**Figure 4E**). The two *BdLM1* knockout transformants, *ΔBdLM1*-1 and *ΔBdLM1*-2, were selected for further study. We also generated two complementary *BdLM1* transformants (C-1 and C-2).

Subsequently, we assessed the growth, conidia production, and conidial germination of the wild type (WT), *BdLM1* knockout mutants, and complementary transformants. The two *BdLM1* knockout mutants exhibited significantly faster growth than the WT and complementary transformants (**Figures 4F, G**). Additionally, the two knockout mutants produced less melanin in 5-d cultures but a similar quantity in 10-d cultures with the WT and complementary strains (**Figure S1**), indicating that the loss of *BdLM1* delayed melanin production in *B*. *dothidea*. However, no significant differences in the formation of pycnidia, the production of conidia, or the germination rates of conidia were observed between the knockout mutants, WT, and complementary strains on potato dextrose agar (PDA) (**Figures 4F, H, I**).

**Figure 5:**
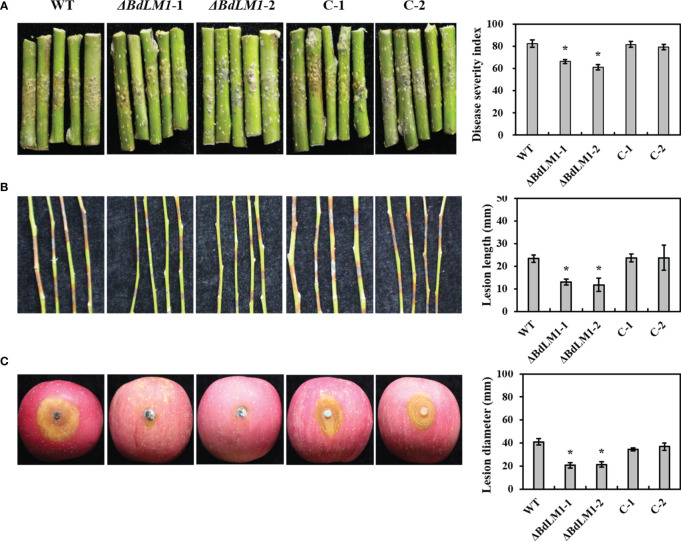
*BdLM1* knockout transformants of *Botryosphaeria dothidea* show reduced virulence on apple shoots and fruit. **(A)** Symptoms and disease severity on intact shoots inoculated with the wild type (WT), knockout transformants, and complementary strains measured 30 d after inoculation; Disease severity was recorded on a scale from 0 to 4 based on the number of warts on inoculation sites using the method described by Dong et al. (2021). The disease severity index (DSI) was calculated through the formula: [sum (class frequency × score of rating class)]/[(total number of inoculation site) × (maximal disease index)] × 100. **(B)** Symptoms and lesion length on wounded shoots measured 5 d after inoculation; **(C)** Symptoms and lesion length on wounded apple fruit measured 2 d after inoculation. Five shoots and three apple fruit were used for each treatment and the entire experiment was repeated once. Error bars represent standard errors calculated from six replicates. The asterisk indicates the significant difference according to the Student’s t-test (*P <0.05).

To examine the effect of *BdLM1* on pathogenicity, we inoculated wounded and intact apple shoots and wounded fruits with the WT and its transformants. All tested isolates produced lesions on apple (**Figure 5**). However, the *BdLM1* knockout mutants displayed a significant decrease in disease severity index on intact apple shoots by 20% (**Figure 5A**), in lesion length on wounded detached apple shoots by 40% (**Figure 5B**), and in lesion size on wounded apple fruit by 40% (**Figure 5C**). These results indicate that *BdLM1* is required for the full virulence of *B*. *dothidea*.

### 3.4 BdLM1 suppresses the immunity of *N. benthamiana*

**Figure 6:**
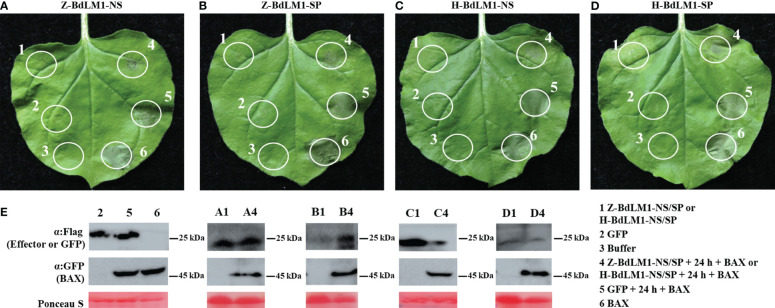
BdLM1 suppresses cell death triggered by BAX in *Nicotiana benthamiana*. **(A)** Suppression of BAX-triggered programmed cell death (BT-PCD) in *N*. *benthamiana* after infiltration with BdLM1 from ZY7 strain without signal peptide (SP). **(B)** Suppression of BT-PCD in *N*. *benthamiana* after infiltration with BdLM1 from ZY7 strain with SP. **(C)** Suppression of BT-PCD in *N*. *benthamiana* after infiltration with BdLM1 from HTLW03 strain without SP. **(D)** Suppression of BT-PCD in *N*. *benthamiana* after infiltration with BdLM1 from HTLW03 strain with SP. The representative photo was acquired 5 d after the last infiltration. **(E)** Western blotting was used to confirm the expression of GFP, BAX, and BdLM1 in **(A-D)**, and equal loading is indicated by Ponceau S staining. Flag tag was added to BdLM1 or GFP, and GFP tag was added to BAX in this study.

To investigate the function of BdLM1 in pathogen–host interactions, we first determined whether BdLM1 could induce programmed cell death (PCD) or suppress BAX-induced programmed cell death (BT-PCD) through *Agrobacterium*-mediated transient expression in *N*. *benthamiana*. Leaves that were challenged with the BAX protein 24 h after infiltration with BdLM1 with or without SP from isolate ZY7 or HTLW03 did not exhibit symptoms of PCD, while leaves infiltrated with GFP or buffer showed PCD (**Figures 6A–D**). Western blot analysis confirmed the expression of GFP, BAX, and BdLM1 in *N*. *benthamiana* leaves after infiltration (**Figure 6E**).

**Figure 7:**
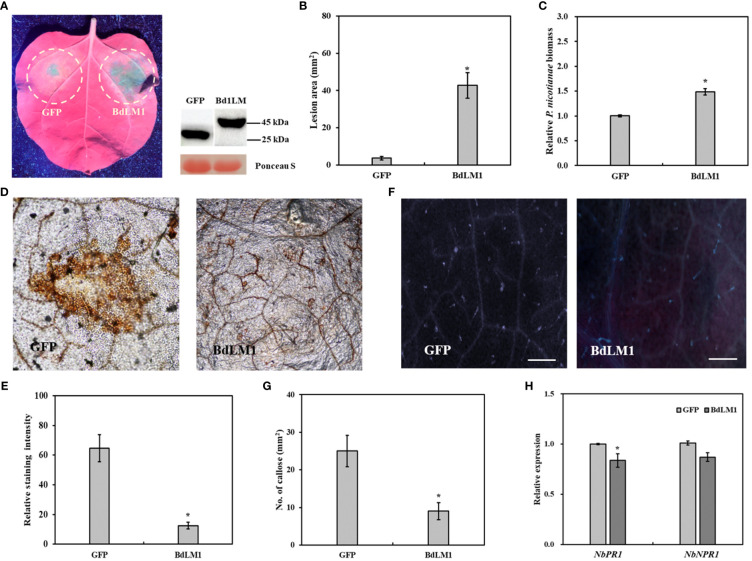
Transient expression of BdLM1 in *Nicotiana benthamiana* inhibits plant immunity and increases *Phytophthora nicotianae* infection. **(A)** Symptoms formed on tobacco leaves inoculated with *P*. *nicotianae* mycelial plug 36 h after transient expression of BdLM1. Photographs were taken under ultraviolet lights, lesion area was measured and biomass was assayed at 60 h post-inoculation (hpi). BdLM was ligated into the plasmid pSuper with a GFP-tag. **(B)** Lesion area. **(C)** Relative biomass of *P. nicotianae*. *Phytophthora nicotianae* biomass in inoculated leaves was determined with quantitative PCR (qPCR) using the *N. benthamiana actin* gene and the *P*. *nicotianae* elongation factor (*EF1α*) gene as internal controls. The data are the averages (and standard errors) of the values from two independent biological replicates. The asterisk indicates the significant difference according to the Student’s t-test (*P <0.05). **(D)** The reactive oxygen burst 48 h after transient expression of GFP or BdLM1. Bars = 200 µm. **(E)** Quantification of DAB staining using ImageJ software. The data are the averages (and standard errors) of the values from two independent biological replicates. The asterisk indicates significant differences according to the Student’s t-test (*P <.05). **(F)** Callose accumulation 48 h after transient expression of GFP or BdLM1. **(G)** Statistical results of callose formation in *N*. *benthamiana* leaves. The data are the averages (and standard errors) of the values from two independent biological replicates. The asterisk indicates significant difference according to the Student’s t-test (*P <0.05). Bars = 200 µm. **(H)** Expression levels of pathogenesis-related (PR) genes in *N*. *benthamiana* 48 h after transient expression of BdLM1. The data are the averages (and standard errors) of the values from two independent biological replicates. The asterisk indicates the significant difference according to the Student’s t-test (*P <0.05).

We further investigated the effects of BdLM1 on the infection of *Phytophthora nicotianae* in the leaves of *N*. *benthamiana*. Leaves transiently expressing BdLM1 showed significantly bigger lesions (**Figures 7A, B**) and a significantly higher relative *P*. *nicotianae* biomass than leaves expressing GFP (**Figure 7C**). Moreover, DAB staining showed a significantly lower level of H_2_O_2_ accumulation in *N*. *benthamiana* tissue inoculated with *P*. *nicotianae* after infiltration with BdLM1 than with GFP (**Figures 7D, E**). Meanwhile, a significant lower quantity of callose deposition was observed in *N*. *benthamiana* tissues infiltrated with BdLM1 than in those infiltrated with GFP (**Figures 7F, G**). In addition, the qRT-PCR assay indicated the downregulated expression of the pathogenesis-related genes *NbPR1* and *NbNPR1* in *N*. *benthamiana* transiently expressing BdLM1 (**Figure 7H**). These results indicate that BdLM1 inhibits plant immunity and promotes *P*. *nicotianae* infection.

Furthermore, we investigated the localization of BdLM1 by fusing the synthetic green fluorescent protein (sGFP) to the C-terminus of *BdLM1*. We infiltrated *Agrobacterium tumefaciens* cells carrying BdLM-sGFP into *N*. *benthamiana* leaves and observed them under a laser confocal microscope. BdLM1 from ZY7 or HTLW03, with or without SP, localized to the nucleus and cytoplasmic membrane of *N*. *benthamiana* (**Figure S2**). These results indicate that BdLM1 probably localizes to nucleus and cytoplasmic membrane of *N*. *benthamiana*.

### 3.5 BdLM1 binds chitin, suppresses reactive oxygen species (ROS) production, and protects hyphae against chitinase hydrolysis

**Figure 8:**
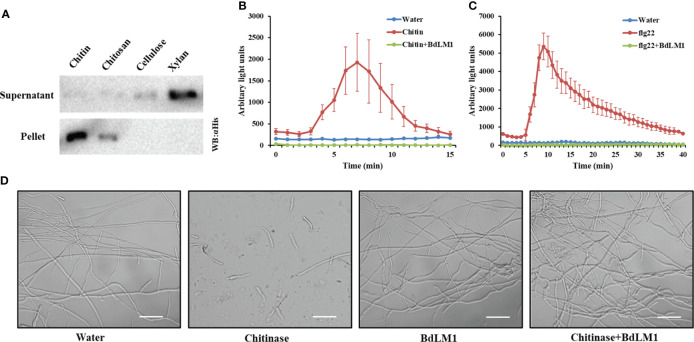
BdLM1 binds chitin, suppresses chitin- and flg22-induced immune responses, and protects the hyphal growth of *Fusarium oxysporum* against chitinase hydrolysis. **(A)** BdLM1 binds chitin and chitosan. *Escherichia coli*-produced BdLM1 was first incubated with chitin, chitosan, cellulose, and xylan for 6 (h) After centrifugation, pellets and supernatants were analyzed using polyacrylamide gel electrophoresis followed by Western blot. **(B)** BdLM1 inhibited the reactive oxygen species (ROS) burst induced by chitin in *N*. *benthamiana*. ROS production in Leaf disks of *N*. *benthamiana* after the addition of 40 µM chitin with or without pre-incubation with 50 µM BdLM1 for 2 (h) The Figure is representative of two independent experiments with similar results. Error bars represent standard errors from four replicates. **(C)** BdLM1 inhibited ROS burst induced by flg22 in *N. benthamiana*. Production of ROS in leaf discs of *N. benthamiana* after the addition of 1 µM flg22 with or without pre-incubation with 50 µM BdLM1 for two hours. The Figure is representative of two independent experiments with similar results. Error bars represent standard errors from four biological replicates. **(D)** BdLM1 protects the hyphal growth of *Fusarium oxysporum* f.sp. *lycopersicum* against chitinase hydrolysis. Micrographs of *F*. *oxysporum* f.sp. *lycopersicum* grown *in vitro* with or without 2 h preincubation with *B*. *dothidea* BdLM1, followed by the addition of chitinase or water. Microscopic pictures were taken approximately 4 h after chitinase addition. Bars = 50 μm.

Previous studies have shown that effectors containing the LysM motif in fungal plant pathogens bind chitin to inhibit plant immunity (de Jonge et al., 2010; Takahara et al., 2016). To investigate how BdLM1 contributes to *B*. *dothidea* virulence during colonization, we first evaluated its substrate-binding characteristics using a polysaccharide precipitation assay following the methods described by Kombrink et al. (2017). The heterologously expressed BdLM1 protein in *Escherichia coli* bound chitin beads and slightly bound chitosan but not the plant cell wall polymers cellulose or xylan (**Figure 8A**).

Previously, LysM effectors from various fungal plant pathogens have been shown to suppress the chitin-induced ROS production of *N*. *benthamiana* leaf disks and have the ability to perturb chitin-induced host immune responses (de Jonge et al., 2010; Tian et al., 2021). To determine whether BdLM1 has this ability, the occurrence of ROS burst induced by chitin or flg22 was assessed in *N*. *benthamiana* leaf disks. This was done by treating the leaf disks with 40 μM chitin or 1 μM flg22, with or without the effector protein BdLM1, as previously demonstrated (de Jonge et al., 2010). Remarkably, pre-incubation of 40 μM chitin or 1 μM flg22 with 50 μM BdLM1 prior to the addition to leaf disks led to a significant reduction of the ROS burst (**Figures 8B, C**), demonstrating its ability to suppress plant immune responses induced by chitin and flg22.

Some LysM proteins have been shown to protect fungal hyphae against chitinase hydrolysis (Marshall et al., 2011; Tian et al., 2021). To evaluate the possible role of BdLM1 in hyphal protection, its ability to protect the hyphae of *Fusarium oxysporum* f. sp. *lycopersicum* was tested. As expected, while the addition of chitinase dramatically hydrolyzed *F*. *oxysporum* hyphae, BdLM1 protected the hyphae from hydrolysis by chitinases from *Streptomyces griseus* (**Figure 8D**).

## 4 Discussion

Although LysM effectors have been extensively characterized in fungal pathogens causing herbaceous plant diseases (Buist et al., 2008; Kombrink et al., 2011), they have rarely been reported in the woody plant fungal pathogen *B. dothidea*, which is well known to cause significant economic losses in agriculture (Guo et al., 2009; Marsberg et al., 2017). Based on bioinformatics analysis, we identified five candidate LysM effectors in *B*. *dothidea*, four of which are closely related to known LysM effectors, such as Mg3LysM, Slp1, and LtLysm. Interestingly, BdLM1, different from those previously studied LysM effectors, is phylogenetically clustered in one group with LysM effectors from *Macrophomina phaseolina*, *Neofusicoccum parvum*, and *Lasiodiplodia theobromae*, among others. The functions of the LysM protein in this group have not yet been documented. This study focused on characterizing the LysM protein BdLM1 from *B*. *dothidea* through gene knockout and infiltration expression. Our results revealed that *BdLM1* knockout mutants showed a significant decrease in virulence in apple. Additionally, BdLM1 suppressed BT-PCD, reduced H_2_O_2_ accumulation and callose deposition, promoted the infection of *P*. *nicotianae*, and significantly downregulated the plant pathogenesis-related gene *PR1* in *N*. *benthamiana*. Furthermore, BdLM1 bound chitin, suppressed plant immunity induced by chitin and flg22 and protected fungal hyphae against chitinase hydrolysis. Our results suggest that the LysM effector BdLM1 plays a crucial role in the full virulence of *B*. *dothidea* and in suppressing plant immunity.

Previously characterized LysM effectors is typically induced during the early stages when pathogens need to evade recognition by the host for successful tissue colonization (Fradin and Thomma, 2006). Similarly, BdLM1 expression peaked at about 6 hpi on wounded apple fruit during early infection. In contrast, its expression is reduced at 1 dpi when the *B*. *dothidea* infection site is generated (Dong et al., 2021) and peaks around 20 dpi on intact shoots during mid to late infection while the infecting hypha penetrates the phellem in the second layer and expands in the phelloderm (Dong et al., 2021). The latter expression pattern is similar to that of the soil-borne vascular plant pathogen *V*. *dahliae*, with the expression of Vd2LysM peaking at about 1 week after inoculation, during xylem colonization, before wilting and the appearance of necrosis (Kombrink et al., 2017). Thus, BdLM1 has variable expression patterns in different apple tissues, including fruit and shoots.

Several LysM proteins have been identified in a single fungal species, such as *M*. *graminicola*, *V*. *dahliae*, and *Penicillium expansum* (Marshall et al., 2011; Kombrink et al., 2017; Levin et al., 2017). In the three LysM effectors of *M*. *graminicola*, only Mg3LysM knockout strains were dramatically changed, including loss of pathogenicity in leaf and asexual sporulation (Marshall et al., 2011). Only lineage-specific Vd2LysM of strain VdLs17 in four LysM effectors from *V*. *dahliae* was required for full virulence in tomatoes (Kombrink et al., 2017). Additionally, four putative PeLysM effectors do not contribute to the virulence of *P*. *expansum* and PeLysM3 has a potential role in growth processes. Similarly, TAL6 in *T*. *atroviride* has been illustrated to be involved in self-signaling processes during fungal growth (Levin et al., 2017). Thus, only some of the LysM effectors from one fungus contribute to virulence. In this study, BdLM1 was required for full virulence of *B. dothidea*, affected penetration and extension, and was involved in mycelial growth of *B*. *dothidea*.

LysM effectors have been shown to affect the chitin-induced plant immune system by either binding chitin or protecting fungal hyphae against chitinase (Marshall et al., 2011; Mentlak et al., 2012; Takahara et al., 2016; Kombrink et al., 2017). Similar to *V*. *dahliae* Vd2LysM, *R*. *irregularis* RiSLM, *M*. *graminicola* Mg1LysM, Mgx1LysM, and Mg3LysM (Marshall et al., 2011; Mentlak et al., 2012; Takahara et al., 2016; Kombrink et al., 2017), BdLM1 can protect hyphae against chitinase hydrolysis (**Figure S3**), but *C*. *fulvum* Ecp6, *M*. *oryzae* Slp1, and *C*. *higginsianum* ChELP1 and ChELP2 do not possess such activity (de Jonge et al., 2010; Mentlak et al., 2012; Takahara et al., 2016). It has been reported that this ability is not determined by LysMs number in proteins but by chitin-induced polymerization, which leads to contiguous LysM effector filaments anchored to chitin in the cell wall of fungi to protect them (Sánchez-Vallet et al., 2020; Tian et al., 2021). Previous studies have shown that the expression of the apple LysM protein MdCERK1-2 is induced by *B*. *dothidea* and that MdCERK1-2 and MdCERK1 can bind chitin, suggesting that MdCERK1-2 and MdCERK1 may play a role in apple immune defense responses as a PRR (Zhou et al., 2018; Chen et al., 2020). Thus, we suppose that BdLM1 may compete with LysM proteins MdCERK1-2 and MdCERK1 for chitin binding in apple, leading to the suppression of plant immunity (**Figure S3**). Interestingly, unlike previous findings with Ecp6 (de Jonge et al., 2010), BdLM1 inhibited flg22-induced plant immunity (**Figure S3**). The core effector necrosis-inducing secreted protein 1 (NIS1) of multiple pathogens could inhibit ROS triggered by both chitin and flg22 through commonly interacting with the PRR-associated kinases BAK1 and BIK1 (Irieda et al., 2018). Meanwhile, BdLM1 also inhibited BT-PCD. Based on these results, it appears that BdLM1 may play a broad role in suppressing plant immunity probably through interaction with BAK1 or BIK1 (Wang et al., 2022; Liu and Tang, 2023).

LysM effectors are characterized by one to several LysM domains, but many have two or three LysM domains (Marshall et al., 2011; Mentlak et al., 2012; Takahara et al., 2016; Levin et al., 2017; Harishchandra et al., 2020). Similar to Mg1LysM and MgxLysM (Marshall et al., 2011), BdLM1 contains a single LysM domain. For the LysM effectors containing only one LysM domain, protein interactions have revealed that two monomers of Mg1LysM or MgxLysM form a chitin-independent homodimer through the β-sheet at the N-terminus of Mg1LysM (Sánchez-Vallet et al., 2020; Tian et al., 2021). Furthermore, Mg1LysM homodimers have been reported to undergo ligand-induced polymerization in the presence of chitin and then develop a polymeric structure that can protect fungal cell walls (Sánchez-Vallet et al., 2020). Collectively, we suspect that the woody fungal pathogen source LysM effector differs from other characterized LysM effectors. Further structural analyses of BdLM1 and the mechanisms of its interaction with other LysM effector proteins in *B*. *dothidea* will be conducive.

## 5 Conclusion

In this study, BdLM1 from the woody plant pathogen fungus *B. dothidea* was shown to be a LysM effector. BdLM1 showed different expression patterns on wounded apple fruit and intact shoots and was required for the full virulence of *B*. *dothidea*. BdLM1 inhibited plant immunity induced by the mouse protein BAX, chitin, and flg22. BdLM1 decreased H_2_O_2_ accumulation and callose deposition, and downregulated resistant gene expression in *N*. *benthamiana*. Furthermore, BdLM1 bound chitin and protected fungal hyphae against degradation by chitinase. These findings indicate that the LysM effector BdLM1 contributes to the full virulence of *B*. *dothidea*, inhibits plant immunity induced by various factors, and has a dual function in inhibiting chitin-triggered plant immunity by binding chitin and protecting fungal hyphae against chitinase hydrolysis.

## Data availability statement

The original contributions presented in the study are included in the article/**Supplementary Material**, further inquiries can be directed to the corresponding author.

## Author contributions

HZ: Data curation, Methodology, Validation, Writing – original draft, Writing – review & editing. S-HW: Data curation, Investigation, Methodology, Writing – original draft. P-HL: Data curation, Investigation, Methodology, Writing – original draft. L-YL: Data curation, Investigation, Methodology, Writing – original draft. XY: Investigation, Methodology, Writing – original draft. C-JZ: Investigation, Methodology, Writing – original draft. L-YG: Writing – review & editing. DW: Methodology, Writing – review & editing. X-QZ: Conceptualization, Validation, Visualization, Writing – review & editing, Funding acquisition, Investigation, Resources, Supervision.

## References

[B1] Boller T. He S. (2009). Innate immunity in plants: An arms race between pattern recognition receptors in plants and effectors in microbial pathogens. Science 324, 742–744. doi: 10.1126/science.1171647 19423812 PMC2729760

[B2] Bozsoki Z. Cheng J. Feng F. Gysel K. Vinther M. Andersen K. R.. (2017). Receptor-mediated chitin perception in legume roots is functionally separable from Nod factor perception. Proc. Natl. Acad. Sci. 114 (38), E8118–E8127. doi: 10.1073/pnas.1706795114 28874587 PMC5617283

[B3] Buist G. Steen A. Kok J. Kuipers O. P. (2008). LysM, a widely distributed protein motif for binding to (peptido) glycans. Mol. Microbiol. 68, 838–847. doi: 10.1111/j.1365-2958.2008.06211.x 18430080

[B4] Cen K. Li B. Lu Y. Zhang S. Wang C. (2017). Divergent LysM effectors contribute to the virulence of *Beauveria bassiana* by evasion of insect immune defenses. PloS Pathog. 13 (9), e1006604. doi: 10.1371/journal.ppat.1006604 28873459 PMC5600412

[B5] Chen Q. Dong C. Sun X. Zhang Y. Dai H. Bai S. (2020). Overexpression of an apple LysM-containing protein gene, MdCERK1–2, confers improved resistance to the pathogenic fungus, *Alternaria alternata*, in *Nicotiana benthamiana* . BMC Plant Biol. 20 (1):146. doi: 10.1186/s12870-020-02361-z 32268888 PMC7386173

[B6] de Jonge R. Thomma B. P. H. J. (2009). Fungal LysM effectors: extinguishers of host immunity? Trend Microbiol. 17, 151–157. doi: 10.1016/j.tim.2009.01.002 19299132

[B7] de Jonge R. van Esse H. P. Kombrink A. Shinya T. Desaki Y. Bours R.. (2010). Conserved fungal LysM effector Ecp6 prevents chitin-triggered immunity in plants. Science 329 (5994), 953. doi: 10.1126/science.1190859 20724636

[B8] Dong B. Guo L. (2020). An efficient gene disruption method for the woody plant pathogen *Botryosphaeria dothidea* . BMC Biotechnol. 20, 1–9. doi: 10.1186/s12896-020-00608-z 32138699 PMC7059327

[B9] Dong B. Zhu X. Fan J. Guo L. (2021). The cutinase bdo_10846 play an important role in the virulence of *Botryosphaeria dothidea* and in inducing the wart symptom on apple plant. Int. J. Mol. Sci. 22, 1910. doi: 10.3390/ijms22041910 33673023 PMC7918748

[B10] Fradin E. F. Thomma B. P. H. J. (2006). Physiology and molecular aspects of *Verticillium* wilt diseases caused by V. dahliae and *V. albo-atrum* . Mol. Plant Pathol. 7, 71–86. doi: 10.1111/j.1364-3703.2006.00323.x 20507429

[B11] Garvey K. J. Saedi M. S. Ito J. (1986). Nucleotide sequence of Bacillus phage phi 29 genes 14 and 15: homology of gene 15 with other phage lysozymes. Nucleic. Acids Res. 14, 10001–10008. doi: 10.1093/nar/14.24.10001 3027653 PMC341351

[B12] Guo L. Li J. Li B. Zhang X. Zhou Z. Li G.. (2009). Investigations on the occurrence and chemical control of *Botryosphaeria* canker of apple in China. Plant Prot. 35, 120–123. doi: 10.3969/j.issn.0529-1542.2009.04.027

[B13] Harishchandra D. L. Zhang W. Li X. Chethana K. W. T. Hyde K. D. Brooks S.. (2020). A LysM Domain-Containing protein LtLysM1is important for vegetative growth and pathogenesis in woody plant pathogen *Lasiodiplodia theobromae* . Plant Pathol. J. 36 (4), 323–334. doi: 10.5423/PPJ.OA.05.2020.0084 32788891 PMC7403516

[B14] Hu W. Luo H. Yang Y. Wang Q. Hong N. Wang G.. (2019). Comprehensive analysis of full genome sequence and Bd-milRNA/target mRNAs to discover the mechanism of hypovirulence in *Botryosphaeria dothidea* strains on pear infection with BdCV1 and BdPV1. IMA Fungus. 10, 3–28. doi: 10.1186/s43008-019-0008-4 32647612 PMC7325678

[B15] Irieda H. Inoue Y. Mori M. Yamada K. Oshikawa Y. Saitoh H.. (2018). Conserved fungal effector suppresses PAMP-triggered immunity by targeting plant immune kinases. PNAS. 16:496–505. doi: 10.1073/pnas.1807297116 PMC6329965 30584105

[B16] Jones J. D. Dangl J. L. (2006). The plant immune system. Nature 444, 323–329. doi: 10.1038/nature05286 17108957

[B17] Kombrink A. Rovenich H. Shi-Kunne X. Rojas-Padilla E. van den Berg G. C. M. Domazakis E.. (2017). *Verticillium dahliae* LysM effectors differentially contribute to virulence on plant hosts. Mol. Plant Pathol. 18 (4), 596–608. doi: 10.1111/mpp.12520 27911046 PMC6638240

[B18] Kombrink A. Sánchez-Vallet A. Thomma B. P. H. J. (2011). The role of chitin detection in plant-pathogen interactions. Microbes Infect. 13, 1168–1176. doi: 10.1016/j.micinf.2011.07.010 21856436

[B19] Lee W. S. Rudd J. J. Hammond-Kosack K. E. Kanyuka K. (2014). *Mycosphaerella graminicola* LysM effector-mediated stealth pathogenesis subverts recognition through both CERK1 and CEBiP homologues in wheat. MPMI 27, 236–243. doi: 10.1094/MPMI-07-13-0201-R 24073880

[B20] Levin E. Ballester A. R. Raphael G. Feigenberg O. Liu Y. Norelli J.. (2017). Identification and characterization of LysM effectors in *Penicillium expansum* . PloS One 10), e0186023. doi: 10.1371/journal.pone.0186023 PMC5662087 29084256

[B21] Li W. Wang B. Wu J. Lu G. Hu Y. Zhang X.. (2009). The *Magnaporthe oryzae* avirulence gene AvrPiz-t encodes a predicted secreted protein that triggers the immunity in rice mediated by the blast resistance gene Piz-t. Mol. Plant-Microbe. 22, 411–420. doi: 10.1094/mpmi-22-4-0411 19271956

[B22] Liang K. Lan J. Wang B. Liu Y. Lu Q. Liu P. (2021). High-quality genome resource of the pathogen of *Botryosphaeria dothidea* causing kiwifruit soft rot. PhytoFrontiers 1, 123–125. doi: 10.1094/PHYTOFR-07-20-0006-A 33090063

[B23] Liu Z. Lian S. Li B. Lu H. Dong X. Wang C. (2016). Draft genome sequence of *Botryosphaeria dothidea*, the pathogen of apple ring rot. Genome Announce. 4, e01142–e01116. doi: 10.1128/genomeA.01142-16 PMC5084859 27789635

[B24] Liu J. Tang D. (2023). Plant immunity research in China. Phytopathol. Res. 5, 37. doi: 10.1186/s42483-023-00196-8

[B25] Livak K. J. Schmittgen T. D. (2001). Analysis of relative gene expression data using real-time quantitative PCR and the 2^–ΔΔCT^ Method. Methods 25 (4), 402–408. doi: 10.1006/meth.2001.1262 11846609

[B26] Marsberg A. Kemler M. Jami F. Nagel J. H. Postma-Smidt A. Naidoo S.. (2017). *Botryosphaeria dothidea*: A talent pathogen of global importance to woody plant health. Mol. Plant Pathol. 18, 477–488. doi: 10.1111/mpp.12495 27682468 PMC6638292

[B27] Marshall R. Kombrink A. Motteram J. Loza-Reyes E. Lucas J. Hammond-Kosack K. E.. (2011). Analysis of two in planta expressed LysM effector homologs from the fungus *Mycosphaerella graminicola* reveals novel functional properties and varying contributions to virulence on wheat. Plant Physiol. 156 (2), 756–769. doi: 10.1104/pp.111.176347 21467214 PMC3177273

[B28] Mentlak T. A. Kombrink A. Shinya T. Ryder L. S. Otomo I. Saitoh H.. (2012). Effector-mediated suppression of chitin-triggered immunity by *Magnaporthe oryzae* is necessary for rice blast disease. Plant Cell. 24 (1), 322–335. doi: 10.1105/tpc.111.092957 22267486 PMC3289562

[B29] Miya A. Albert P. Shinya T. Desaki Y. Ichimura K. Shirasu K.. (2007). CERK1, a LysM receptor kinase, is essential for chitin elicitor signaling in *Arabidopsis.* Proc. Natl. Acad. Sci. 104, 19613–19618. doi: 10.1073/pnas.0705147104 PMC2148337 18042724

[B30] Rao Y. Mei L. Zhang L. Jiang H. Ma L. Wang Y. (2021). Genome sequence resource of *Botryosphaeria dothidea* CK16, a fungal pathogen causing Chinese hickory trunk canker disease. Plant Dis. 105, 3282–3284. doi: 10.1094/PDIS-02-21-0254-A 33761770

[B31] Rovenich H. Zuccaro A. Thomma B. P. H. J. (2016). Convergent evolution of filamentous microbes towards evasion of glycan-triggered immunity. New Phytol. 212 (4), 896–901. doi: 10.1111/nph.14064 27329426

[B32] Sánchez-Vallet A. Saleem-Batcha R. Kombrink A. Hansen G. Mesters J. R. (2013). Fungal effector Ecp6 outcompetes host immune receptor for chitin binding through intrachain LysM dimerization. Elife 2, e00790. doi: 10.7554/eLife.00790.013 23840930 PMC3700227

[B33] Sánchez-Vallet A. Tian H. Rodriguez-Moreno L. Valkenburg D. J. Saleem-Batcha R. Wawra S.. (2020). A secreted LysM effector protects fungal hyphae through chitin-dependent homodimer polymerization. PloS Pathog. 16, 1–21. doi: 10.1371/journal.ppat.1008652 PMC7337405 32574207

[B34] Shimizu T. Nakano T. Takamizawa D. Desaki Y. Ishii-Minami N. Nishizawa Y.. (2010). Two LysM receptor molecules, CEBiP and OsCERK1, cooperatively regulate chitin elicitor signaling in rice. Plant J. 64, 204–214. doi: 10.1111/j.1365-313x.2010.04324.x 21070404 PMC2996852

[B35] Takahara H. Hacquard S. Kombrink A. Hughes B. Halder V. Robin G. P.. (2016). *Colletotrichum higginsianum* extracellular LysM proteins play dual roles in appressorial function and suppression of chitin-triggered plant immunity. New Phytol. 211 (4), 1323–1337. doi: 10.1111/nph.13994 27174033

[B36] Tang W. Ding Z. Zhou Z. Wang Y. Guo L. (2012). Phylogenetic and pathogenic analyses show that the causal agent of apple ring rot in China is *Botryosphaeria dothidea* . Plant Dis. 96, 486–496. doi: 10.1094/PDIS-08-11-0635 30727432

[B37] Thomma B. P. H. J. Nurnberger T. Joosten M. H. A. J. (2011). Of PAMPs and effectors: the blurred PTI-ETI dichotomy. Plant Cell. 23, 4–15. doi: 10.1105/tpc.110.082602 21278123 PMC3051239

[B38] Tian H. Mackenzie C. I. Rodriguez-Moreno L. Berg G.C.M.V.D. Chen H. Rudd J. J.. (2021). Three LysM effectors of *Zymoseptoria tritici* collectively disarm chitin-triggered plant immunity. Mol. Plant Pathol. 22 (6), 683–693. doi: 10.1111/mpp.13055 33797163 PMC8126183

[B39] Wang B. Liang X. Gleason M. Zhang R. Sun G. (2018). Comparative genomics of *Botryosphaeria dothidea* and *B. kuwatsukai*, causal agents of apple ring rot, reveals both species expansion of pathogenicity-related genes and variation in virulence gene content during speciation. Ima Fungus 9 (2), 243–257. doi: 10.5598/imafungus.2018.09.02.02 30622881 PMC6317582

[B40] Wang Y. Pruitt R. N. Nürnberger T. Wang Y. (2022). Evasion of plant immunity by microbial pathogens. Nat. Rev. Microbiol. 20, 449–464. doi: 10.1038/s41579-022-00710-3 35296800

[B41] Wang S. Zhang C. Weng S. Guo L. Zhu X. (2019). Candidate effectors of *Botryosphaeria dothidea* inhibit Bax induced PCD in *Nicotiana benthamiana* and promote the infection of *Phytophthora nicotianae.* Acta Phytopathol. Sin 49 (02), 254–261. doi: 10.13926/j.cnki.apps.000288

[B42] Xiao Z. Li B. Guo L. (2013). The occurrence of sexual stage of *Botryosphaeria dothidea* in apple main producing areas of China. J. Fruit Trees. 30 (06), 1005–1010 + 1109. doi: CNKI:SUN:GSKK.0.2013-06-020

[B43] Yang Q. Huai B. Lu Y. Cai K. Guo J. Zhu X.. (2019). A stripe rust effector Pst18363 targets and stabilizes TaNUDX23 that promotes stripe rust disease. New Phytol. 225 (2), 880–895. doi: 10.1111/nph.16199 31529497

[B44] Yu C. Diao Y. Lu Q. Zhao J. Cui S. Peng C.. (2021). Genome assembly and annotation of *Botryosphaeria dothidea* sdau11-99, a latent pathogen of apple fruit ring rot in China. Plant Dis. 105, 1555–1557. doi: 10.1094/PDIS-06-20-1182-A 33258431

[B45] Zeng T. Rodriguez-Moreno L. Mansurkhodzaev A. Wang P. van den Berg W. Gasciolli V.. (2020). A lysin motif effector subverts chitin-triggered immunity to facilitate arbuscular mycorrhizal symbiosis. New Phytol. 225, 448–460. doi: 10.1111/nph.16245 31596956 PMC6916333

[B46] Zhang C. Wang S. Liang Y. Wen S. Dong B. Ding Z.. (2021). Candidate effectors from *Botryosphaeria dothidea* suppress plant immunity and contribute to virulence. Int. J. Mol. Sci. 22 (2), 552. doi: 10.3390/IJMS22020552 33430504 PMC7826910

[B47] Zhou Z. Tian Y. Cong P. Zhu Y. (2018). Functional characterization of an apple (*Malus x domestica*) LysM domain receptor encoding gene for its role in defense response. Plant Sci. 269, 56–65. doi: 10.1016/j.plantsci.2018.01.006 29606217

