# Supplementary material for: LysM protein BdLM1 of Botryosphaeria dothidea plays an important role in full virulence and inhibits plant immunity by binding chitin and protecting hyphae from hydrolysis
Source: Front Plant Sci. 2024 Jan 8;14:1320980. doi: 10.3389/fpls.2023.1320980 (PMC10800735; doi:10.3389/fpls.2023.1320980)
Supplement: Supplementary file 1 [file DataSheet_1.docx]

Supplementary Material

# Supplementary Figures and Tables

## Supplementary Figures


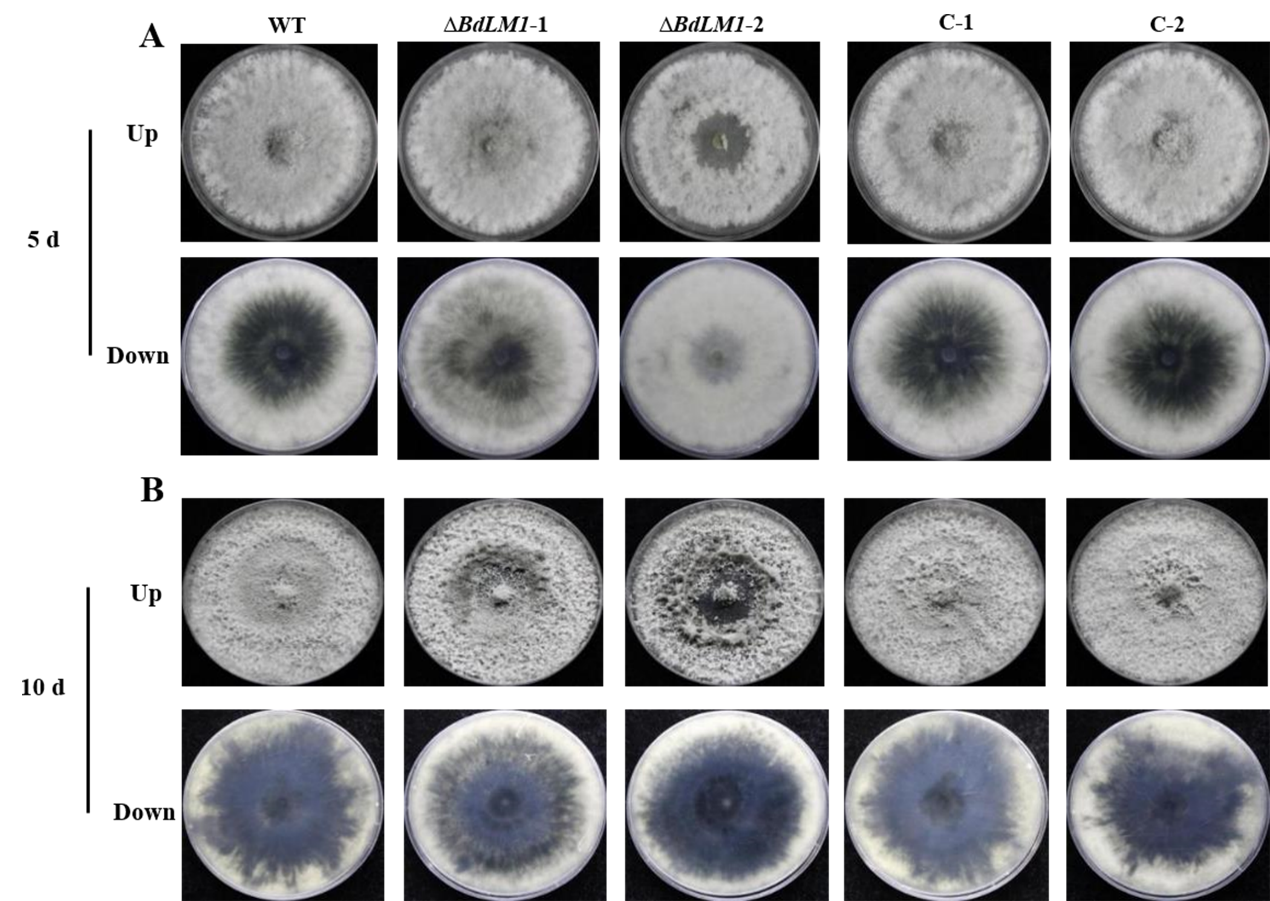


**Supplementary Figure 1. Melanin production of the wild type and *Bd1LM* deletion mutants on PDA media after 5 and 10 d.** (A) Melanin production of the wild type and *Bd1LM* deletion mutants on PDA media after 5 d; (B) Melanin production of the wild type and *Bd1LM* deletion mutants on PDA media after 10 d.


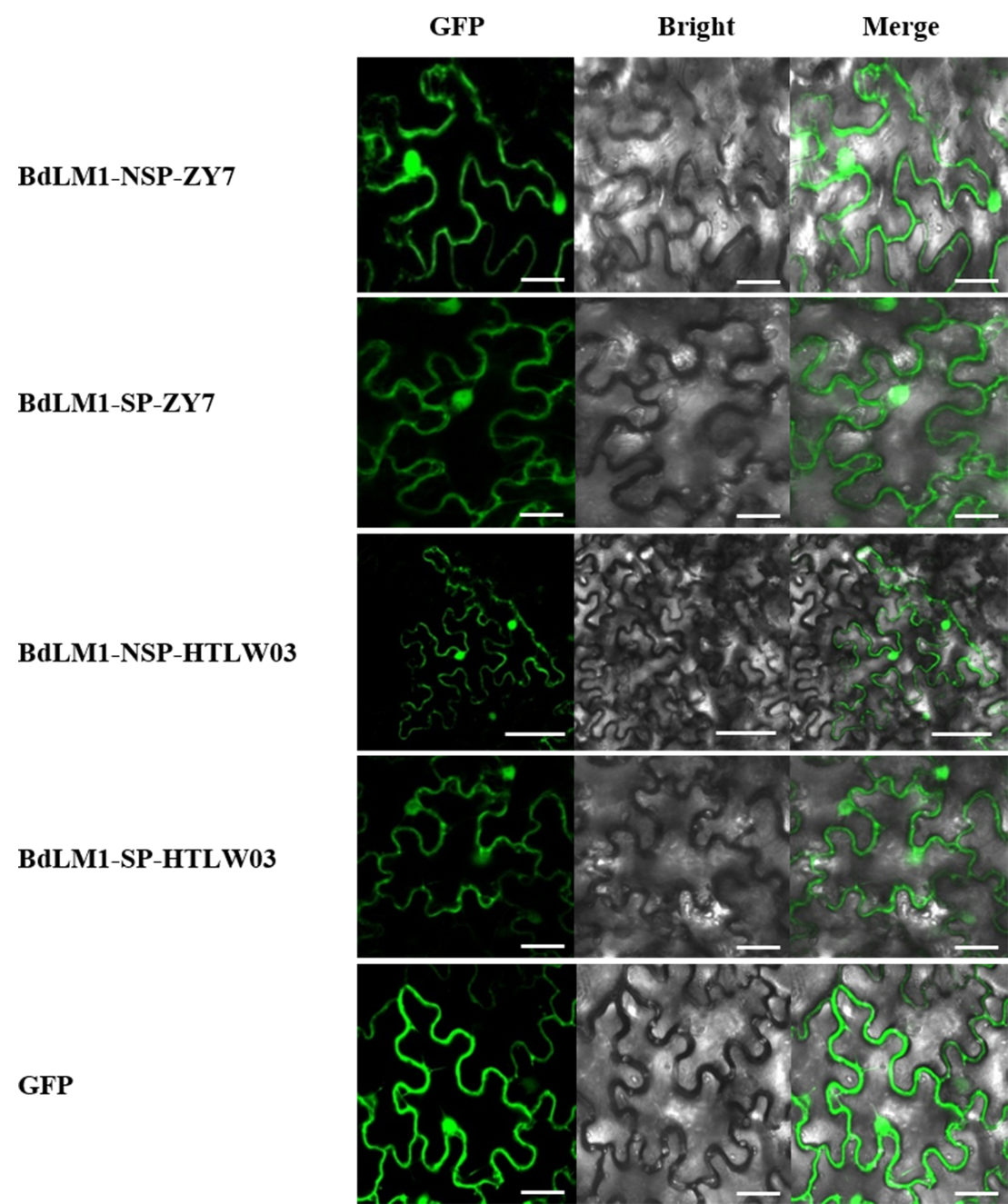


**Supplementary Figure 2. Subcellular localization of BdLM1 in *Nicotiana benthamiana* leaves.** Subcellular localization of BdLM1 with C-terminally green fluorescent protein (GFP)-tagged through transient expression in *N*. *benthamiana* leaves. The fluorescence was scanned using a Leica TCS SP8 confocal microscopy system. Bars = 20 μm. NSP: non-signal peptide; SP: signal peptide.


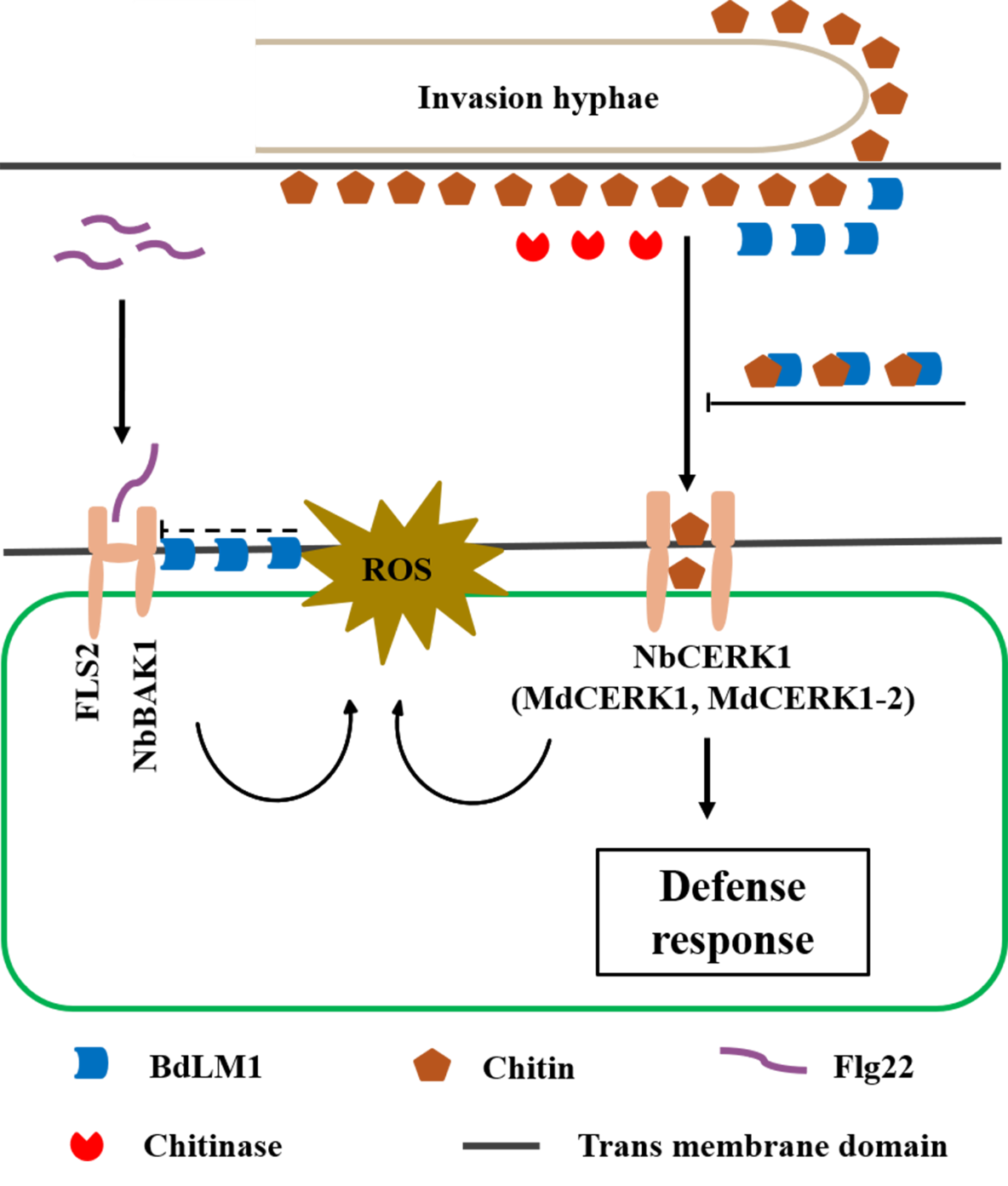


**Supplementary Figure 3. A model for the roles of BdLM1 in *Botryosphaeria dothidea* in host infection.** Upon invading the host, BdLM1 protects the hyphae from plant hydrolysis. At the same time, BdLM1 can bind chitin by competing with host receptors to escape from host recognition, leading to a decrease in reactive oxygen species (ROS) production and the subsequent downregulation of pathogenesis-related (PR) genes. BdLM1 also suppresses ROS activated by flg22, perhaps through interacting with conservative components, such as BAK1.

## Supplementary Tables

**Supplementary Table 1. Primers used in this study.**

| **Description** | **Primer name** | **Sequence (5’-3’)** |
| --- | --- | --- |
| Amplifying 5’ flanking of *BdLM1* | BdLM1-Upstream-F | acggccagtgaattcgagctcAATCCTCAAGTATTGTTCGCTGCAACC |
|  | BdLM1-Upstream-R | ggatccccgggtaccgagctcGTTGAAGATGTTTCGGATAGACGTCGT |
| Amplifying 3’ flanking of *BdLM1* | BdLM1-Downstream-F | gacctgcaggcatgcaagcttTCCAATTCGAGGAAGCATGACGAGAG |
|  | BdLM1-Downstream-R | gaccatgattacgccaagcttTTACGGTTCGCAGATCCAGTACTCGT |
| PCR verification for *BdLM1* knockout transformants | BdLM1-1F | TCTAGAAGTATTCCAGACTTTTCTGCTGAGCA |
|  | BdLM1-1R | ACATATCCACGCCCTCCTACATCGAA |
|  | BdLM1-2F | TCATTGACTGGAGCGAGGCGATGTT |
|  | BdLM1-2R | ACAACTCCACGTCTGGAGACCTCAT |
|  | BdLM1-3F | AGAACCATCTCTGCCGCTCGTTTATT |
|  | BdLM1-3R | TACTCGCGTAAGGCTGTCGTGATGT |
| Amplification for signal peptide of BdLM1 | pSUC2-BdLM1-SP-F | TTTAATTAAGAATTCATGGCCAAGTTCGCCACTCTCCTC |
|  | pSUC2-BdLM1-SP-R | AGGGAGAACCTCGAGGGTAGTGCAGTTGCTGCCATCCTG |
| Amplification for candidate effector gene | pGR107-BdLM1-SP-F | agaactagtggatcccccgggATGGCCAAGTTCGCCACTCTC |
|  | pGR107-BdLM1-SP-R | aaccgttcatcggcggtcgacTTAAGCCTTGACGCACACCCA |
|  | pGR107-BdLM1-NS-F | agaactagtggatcccccgggATGTTCCCTTCCCCGTTCCCT |
|  | pGR107-BdLM1-NS-R | aaccgttcatcggcggtcgacTTAAGCCTTGACGCACACCCA |
|  | pSuper-BdLM1-F | atacaccaaatcgactctagaATGTTCCCTTCCCCGTTCC |
|  | pSuper-BdLM1-R | gcccttgctcaccattctagaAGCCTTGACGCACACCCA |
| Amplification for subcellular localization of BdLM1 | pCam35S-BdLM1-SP-F | ggtacccgggatcctctagaATGGCCAAGTTCGCCACTCTCCTC |
|  | pCam35S-BdLM1-SP-R | cttgctcaccatggtgtcgacTTAAGCCTTGACGCACACCCAGTAATC |
|  | pCam35S-BdLM1-NS-F | ggtacccgggatcctctagaATGTTCCCTTCCCCGTTCCCTTCC |
|  | pCam35S-BdLM1-NS-R | ggtacccgggatcctctagaTTAAGCCTTGACGCACACCCAGTAATC |
| Quantification for expression level of different growth and infection stages | qRT-BdLM1-F | GTTCCGCTCATGCCCAACACCATC |
|  | qRT-BdLM1-R | CCTTGACGCACACCCAGTAATCGG |
| Quantification for the ratio of host to pathogen biomass | BdLM1-F | ATGTTCCCTTCCCCGTTCCCT |
|  | BdLM1-R | TTAAGCCTTGACGCACACCCA |
|  | PnEF1-F | ACTCCAAGAACGACCCTGCTAAGGCAACC |
|  | PnEF1-R | TTCGACGGCTCGAGGATGACCATGCAG |
|  | NbAct-F | ACCATCAATGATCGGAATGG |
|  | NbAct-R | GCTCATCCTATCAGCAATGC |
| Quantification for expression levels of pathogensis-related (PR) genes | NbPR1-F | GACGACCAGGTAGCAGCCTATG |
|  | NbPR1-R | CAACAGCCTTAGCAGCCGTCATG |
|  | NbNPR1-F | TGCAGCAGACGATGTAATGGTGGT |
|  | NbNPR1-R | CTTGTAGACCAAGTTCTGCTCGTG |
|  | NbEF1-F | CTGCCAGCTTTACCTCCCAAGTCA |
|  | NbEF1-R | CCAGAACGCCTGTCGATCTTGGT |
| Amplification for construction of prokaryotic expression vector | pET-SUMO-BdLM1-F | tttattttcagggcgccatggTTCCCTTCCCCGTTCCCT |
|  | pET-SUMO-BdLM1-R | gtggtggtggtggtgctcgagTTAAGCCTTGACGCACACCC |
